# *Echinacea purpurea* L. (Moench) Hemagglutinin Effect on Immune Response In Vivo

**DOI:** 10.3390/plants10050936

**Published:** 2021-05-07

**Authors:** Gabrielė Balčiūnaitė-Murzienė, Zoja Miknienė, Ona Ragažinskienė, Nomeda Juodžiukynienė, Arūnas Savickas, Nijolė Savickienė, Dalia Pangonytė

**Affiliations:** 1 Faculty of Pharmacy, Institute of Pharmaceutical Technologies, Academy of Medicine, Lithuanian University of Health Sciences, Sukileliu Ave. 13, 50162 Kaunas, Lithuania; 2 Large Animal Clinic, Faculty of Veterinary, Veterinary Academy, Lithuanian University of Health Sciences, Tilzes Str. 18, 47181 Kaunas, Lithuania; zoja.mikniene@lsmuni.lt; 3 Kaunas Botanical Garden, Vytautas Magnus University, Z. E. Zilibero Str. 6, 46324 Kaunas, Lithuania; ona.ragazinskiene@vdu.lt; 4 Department of Veterinary Pathobiology, Faculty of Veterinary, Academy of Veterinary, Lithuanian University of Health Sciences, Tilzes Str. 18, 47181 Kaunas, Lithuania; nomeda.juodziukyniene@lsmuni.lt; 5 Department of Drug Technology and Social Pharmacy, Faculty of Pharmacy, Academy of Medicine, Lithuanian University of Health Sciences, Sukileliu Ave. 13, 50162 Kaunas, Lithuania; arunas.savickas@lsmuni.lt; 6 Department of Pharmacognosy, Faculty of Pharmacy, Academy of Medicine, Lithuanian University of Health Sciences, Sukileliu Ave. 13, 50162 Kaunas, Lithuania; nijole.savickiene@lsmuni.lt; 7 Laboratry of Cardiac Pathology, Institute of Cardiology, Lithuanian University of Health Sciences, Sukileliu Ave. 15, 50162 Kaunas, Lithuania; dalia.pangonyte@lsmuni.lt

**Keywords:** *Echinacea purpurea* L. (Moench), lectin, hemagglutinin, LysM, immune response, T lymphocytes, spleen, blood cells

## Abstract

*Echinacea purpurea* L. (Moench) is used in traditional and conventional medicine. However, there is lack of data on the biological activities of primary plant metabolite lectins. The aim of our experiment was to find out how lectin LysM (lysine motif), which was previously purified, affects the immune response in vivo. Eight-week-old BALB/c male mice (n = 15) received four weekly 250 μg/kg peritonial injections of purified *Echinacea purpurea* L. (Moench) roots’ LysM lectin. The control animal group (n = 15) received 50 μL peritoneal injections of fresh *Echinacea purpurea* L. (Moench) root tincture, and the negative control animal group (n = 15) received 50 μL peritoneal injections of physiological solution. At the fifth experimental week, the animals were sedated with carbon dioxide, and later euthanized by cervical dislocation, and then their blood and spleen samples were collected. The leukocytes’ formula and lymphocytes’ count was estimated in blood samples, the T lymphocytes’ density was evaluated in spleen zones. A statistically significant (*p* < 0.05) difference between each group was observed in the leukocytes’ formula (monocytes’ percentage, also little, medium and giant size lymphocytes). The purple coneflower fresh roots’ tincture significantly decreased (*p* < 0.05) the T lymphocytes’ quantity in peritoneal lymphoid sheaths (PALS) compared with the physiological solution injection’s group (*p* < 0.05) and the lectin injection’s group (*p* < 0.001). Meanwhile, lectin injections caused a significant (*p* < 0.01) increase in the T lymphocytes in a spleen PALS zone, compared with the physiological solution and tincture injection’s group. Our data suggests that LysM lectin acts as an immunostimulant, while fresh purple coneflower tincture causes immunosuppression.

## 1. Introduction

*Echinacea purpurea* (L.) Moench is an endemic plant in the U.S. Great Plains and the Canadian prairies of North America. The indigenous traditional healers from North American tribes of Cheyenne, Choctaw, Dakota, Delaware, Fox Kiowa, Montana, Omaha Pawnee, Ponca, Sioux, and Winnebago indicated *Echinacea* preparations for the healing of wounds, skin inflammatory conditions, infectious diseases, coughs and chest conditions, sore throat and thrush, and also as an antidote against snakebites and poisons, and as a pain reliever [1,2]. Even though Echinacea originates in North America, the traditional knowledge of this plant’s beneficial effects was introduced to European settlers and it has spread beyond the Atlantic Ocean. In modern times, the plant’s traditional description was changed from “anti-infective” to immunomodulatory active [3,4]. It is worth to mention that the immunomodulatory activity might be related to antimicrobial and antiviral activities due to the synergistic effect on the host immune system [3,5]. The immune-enhancing effects of *Echinacea purpurea* (L.) Moench preparations are well documented and the underlying mechanisms have been widely investigated [6,7,8,9]. The plant’s important components are caffeic acid derivatives, alkylamides, flavonoids, polysaccharides, lipoproteins and polyacetylenes. Among them, the caffeic acid derivatives and alkylamides were proved to have effects of immunoregulation [10]. At their highest concentration, polysaccharides are typically present in aqueous or fresh pressed juice extracts, while alkylamides (as major constituents) are more likely to be found in ethanolic extracts [10].

Hemagglutinins (lectins) are natural bioactive proteins and glycoproteins that have the capability to specifically bind sugars [11]. These sugar-binding proteins are of non-immune origin, and can agglutinate cells or precipitate glycoconjugates [12]. Previously, numerous lectins have been isolated from various sources such as plants, algae, and fungi. Plants’ lectins are known to have immunomodulatory activity. They are capable of modulating the secretion of cytokines and the production of other immune mediators, such as reactive oxygen species (ROS) and reactive nitrogen intermediates (RNI), in order to improve the host’s resistance to microbial infections [13]. Plants’ lectins have the ability to bind to specific carbohydrate residues, which are present in the membrane of both the bacterial and immune cells of the host. Plants’ lectins are mitogens that have the ability to stimulate lymphocytes to undergo mitosis in a calcium-dependent manner [11]. This stimulation is a result of the interaction between the plants’ lectins and the surface’s sugar moieties present on the surface of lymphocytes. T lymphocytes’ cell proliferation is an essential factor to monitor the different adaptive immune reactions. In a variety of studies, authors used spleen, mainly of mice, to investigate the mitogenic power of lectins. Splenocytes encompass macrophages, dendritic cells, and B and T cells; these splenic populations are capable of generating innate and adaptive immune responses [10].

Lactose-, D-mannose-, and D-galactose-specific glycoproteins (~40 kDa) were extracted and purified from fresh *Echinacea purpurea* (L.) Moench root. The purified glycoproteins were identified by LS-MS/MS as a two LysM (lysine motif) domain that contains lectins. The LysM domain, which contained lectins, demonstrated hemagglutinating activity [14]. There are no data on *Echinacea purpurea* (L.) Moench’s lectin effect on the immune response in vivo. Therefore, in the present study we investigated the effects of *Echinacea purpurea* (L.) Moench (EP) fresh roots’ tincture, and purified (from *Echinacea purpurea* (L.) Moench fresh root) glycoproteins with the LysM domain in a mouse model by analyzing samples of blood and spleen.

## 2. Results

### 2.1. Results of Tincture Qualitative and Quantitative Assessment by HPLC

**Figure 1:**
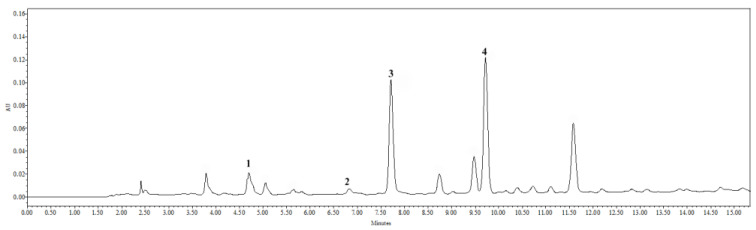
HPLC analysis chromatogram of fresh *Echinacea purpurea* (L.) Moench roots‘ tincture (1:5). Identified phenolic acids as follows: 1—chlorogenic acid, 2—caftaric acid, 3—caffeic acid, and 4—cichoric acid.

The HPLC analysis of 50% (*v*/*v*) ethanolic purple coneflower fresh roots’ tincture (1:5) was done by the method described in the European Pharmacopoeia [15]. The results are represented in Figure 1. Phenolic acids (1—chlorogenic, 2—caftaric, 3—caffeic and 4—cichoric acids) were identified according to their retention time and UV light absorption spectra. These phenolic acids were quantified in equivalents of caffeic acid. Quantitative assessment of the analytes was performed based on the analyte peak area, depending on analyte concentration in the test solution. Table 1 represents formula of the caffeic acid’s calibration curve, correlation coefficient, lowest observed quantity and lowest quantity detected, as well as calculations.

Caffeic acid, 3.78 ± 0.23 μg per one mL tincture, was estimated. Caftaric acid’s quantity in caffeic acid’s equivalents was 1.95 ± 0.10 μg/mL, cichoric acid’s—3.47 ± 0.14 μg/mL and 1.75 ± 0.09 μg/mL of chlorogenic acid’s. However, chlorogenic acid’s quantity was lower than the detectable concentration, so this result is not significant.

Our experiments show that caffeic and chlorogenic acids are dominant in our tincture. These results agree with previous investigations [16,17]. Nonetheless, the quantities of these acids in our tincture were smaller, compared to tinctures that were made from dried *Echinacea purpurea* (L.) Moench roots.

To sum up, we extracted 92.75 ± 34.47 mg of chemical substances from 1 g of purple coneflower’s fresh roots. We have identified 16.37 µg of phenolic compounds, or 0.01% from all extracted dry residue. Our tincture contained higher quantities of non-identified biologically active compounds, such as alkylamides, polysaccharides and lipopolysaccharides, typical to purple coneflower roots.

### 2.2. Animals during Experiment

All 44 experimental animals survived 4 experimental weeks. No behavior changes were noticed. Although, during the second investigation week, the following noticeable changes of animal fur occurred: the hemagglutinin LysM group’s mice coat became greasy, in contrast the tincture group’s mice coat appeared to look healthy and shiny, and animals that received physiological solution did not appear to get any visible coat changes. The lectin group’s mice fur changes might be explained by the nephrotoxicity data of the LysM domain containing EP lectin, which was published previously [12].

### 2.3. Blood Morphology and Cell Count

**Figure 2:**
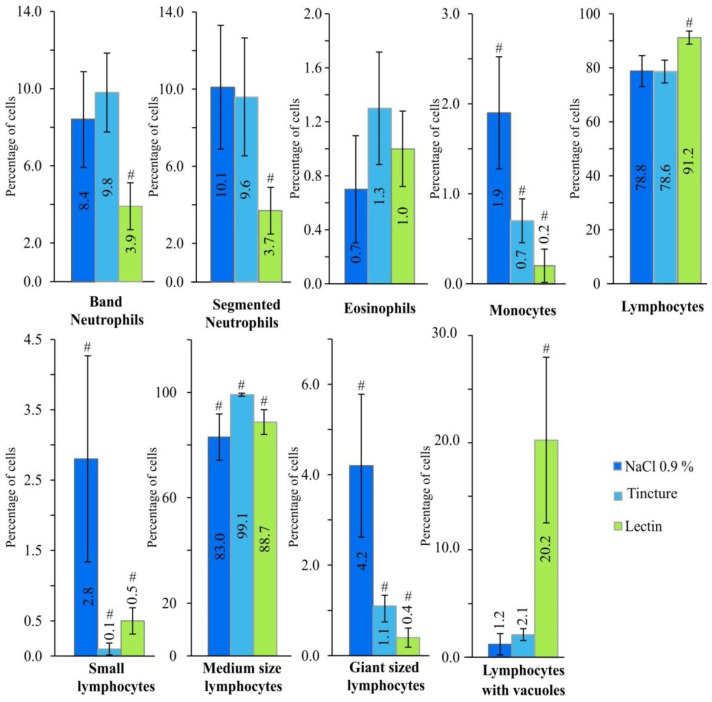
Leukocytes’ formula and white blood cells count comparison after treatment of mice, which was done four times every seven days with NaCl 0.9% 50 μL, EP roots’ tincture 2.5 μL/kg and hemagglutinin’s fraction 2.5 mg/kg. # Statistical significance *p* < 0.05.

Four weeks of treatment with injections of peritoneal EP roots’ tincture and hemagglutinin LysM resulted in significant differences in mice white blood cells counts and leukocytes’ formula between groups, compared to the animal group that received treatment with physiological solution. A statistically significant (*p* < 0.05) difference between each group was observed in the leukocytes’ formula, monocytes’ percentage, as well as little, medium and giant size lymphocytes (Figure 2).

**Figure 3:**
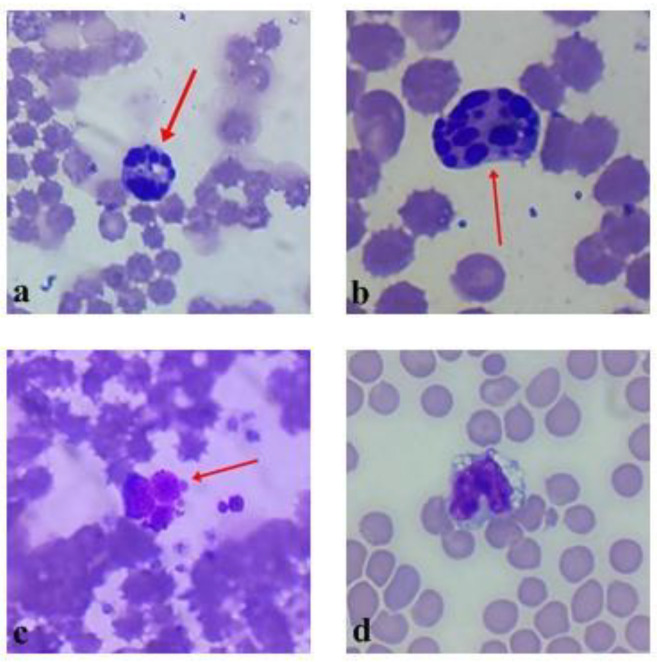
(**a**–**d**) Represent abnormalities of cell that were detected in peripheral blood of mice after treatment with EP hemagglutinin LysM. (**a**–**c**) Represent leucocyte cells with defragmented (apoptotic) nucleus. (**d**) Vacuolated band neutrophil. No cell abnormalities were detected in groups of negative control and EP tincture.

The low number of monocytes in the group of the hemagglutinin LysM could indicate induced inflammatory processes. Foreign proteins might have induced the monocytes’ migration and development to macrophages. In mice, lectin, which was isolated from *Talisia esculenta* (A. St. Hil) seeds, induced an inflammatory peritoneum reaction accompanied by the influx of polymorpho-nuclear leukocytes [18]. The inflammation influenced significant neutrophil and mononuclear cell recruitment. The mononuclear phagocyte system (MPS) plays a role by directly eliminating foreign agents and organizing each different phase of the inflammatory process [19]. As inflammation progresses, circulating monocytes are recruited into the inflamed tissue. Later, they differentiate into inflammatory macrophages in order to phagocytose dead neutrophils and other dead cells to ensure reparation of the tissue [20]. Macrophages are sentinels of the tissue that maintain the integrity of the tissue by eliminating/repairing damaged cells and matrices [21]. This hypothesis could be supported by a drastically increased (statistically significant (*p* < 0.05)) percentage of vacuolated leukocytes and a statistically significant (*p* < 0.05) decrease in the band and segmented neutrophils in the hemagglutinin animal group. Moreover, atypical changes in the lymphocytes and neutrophils were noticed in the hemagglutinin LysM animal group. Some of the cells contained a defragmented nucleus (apoptosis) (Figure 3).

**Figure 4:**
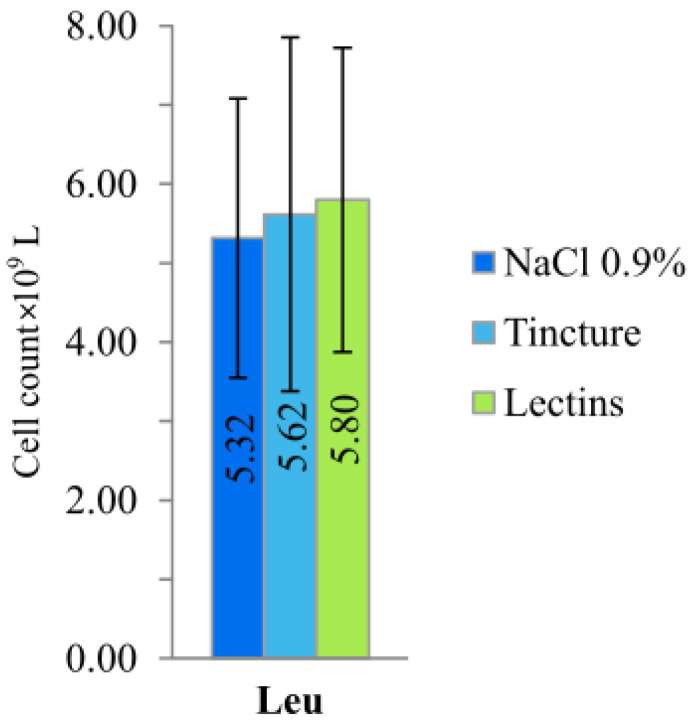
White blood cell count comparison after treatment of the mice for four times every seven days with NaCl 0.9%, EP roots’ tincture and hemagglutinin’s fraction.

Even though there was no statistical significance in the changes in the white blood cell count (Figure 4), immune stimulation was indicated by a statistically significant (*p* < 0.05) increase in lymphocytes in the hemagglutinin LysM animal group. However, the increased number of vacuolated or atypical pyknotic (apoptotic) lymphocytes shows that protein could have toxic effects. This hypothesis is supported by the differences in animal coat appearance and nephrotoxicity of the LysM domain containing EP lectin [14]. The differentiation of macrophages from monocytes occurs in the tissue, in concomitance with the acquisition of a functional phenotype that depends on the microenvironmental signals, thereby accounting for a lot of apparently opposed functions of macrophages [21]. The most discussed current classification of macrophages is based on the M1/M2 paradigm, which is related to their pro- and anti-inflammatory properties [22]. Proposed in the early 21st century, the M1/M2 paradigm states that macrophages can switch their phenotypes from the pro-inflammatory M1 to the anti-inflammatory M2, and vice versa, depending on the needs of the microenvironment, or they can maintain the naïve state M0 in the absence of external signals [23]. Monocytes and macrophages are never isolated in the body. Their phenotype and actions are totally influenced by the surrounding cells and tissue [24].

Since macrophages are derived from monocytes, the percentage of monocytes in the blood may decrease once the nonspecific immune response turns specific. The antigens that are too difficult to digest or too harmful may accumulate in the macrophages by forming vacuoles. In that case, a larger percentage of vacuolated macrophages are found in the blood [25,26]. If the nonspecific immune response is not successful enough to completely remove the antigen from the organism, then antigen-presenting cells (for instance, dendritic cells or macrophages) present a part of the antigen to T-cells, which are located in the lymph nodes. Therefore, the adaptive immune response initiates [27]. Unlike innate immunity, which uses the recognition molecules that are expressed broadly on a large number of cells and acts rapidly after an invading pathogen or toxin is encountered, the adaptive system is composed of small numbers of cells with specificity for any individual antigen. The responding cells of the adaptive system must proliferate after the encountering of the antigen, in order to attain sufficient numbers to arrange an effective response against the antigen [28]. In that case, the percentage of B and T lymphocytes in the blood may increase [29]. It is discovered that, besides their main role (i.e., clearance of parasitic infection), eosinophils have a role in antigen presentation and T-cell regulation, and so the percentage of eosinophils in the blood may increase, in the case of a more intense specific immune response [26]. The results of our study showed the statistically significant (*p* < 0.05) increase in lymphocytes, and no statistical significant increase in eosinophil count in the hemagglutinin’s LysM animal group.

### 2.4. Spleen Immunohistochemical Investigation

**Figure 5:**
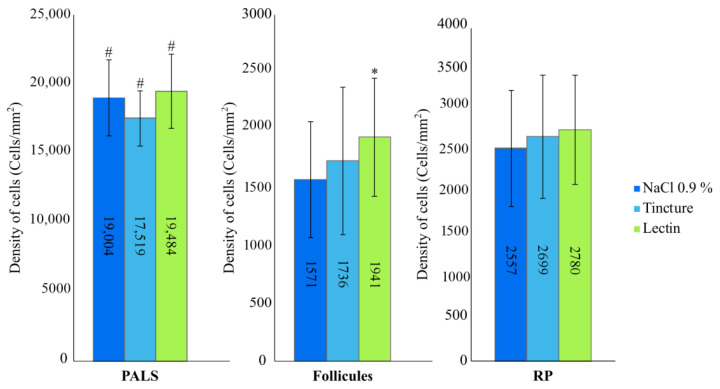
T lymphocytes’ density (cells/mm^2^) distribution in BALB/c mice spleen. PALS, follicles and red pulp (RP). * *p* < 0.01 compared with physiological solution’s and *p* < 0.05 compared with tincture’s group. # Statistical significance *p* < 0.05.

The spleen is the biggest immune system organ, where a specific immune response is generated, as well as where hematopoiesis and erythrocyte deconstruction occurs [30]. The experimental data of the spleen’s immunohistochemical analysis is represented in Figure 5. Purple coneflower fresh roots’ tincture statistically significantly decreased (*p* < 0.05) the T lymphocytes’ quantity in periarterial lymphoid sheaths (PALS), compared with the physiological solution injection group (*p* < 0.05) and the lectin injection group (*p* < 0.001). Meanwhile, lectin injections caused a statistically significant (*p* < 0.01) T lymphocyte increase in the spleen’s PALS zone, compared with the physiological solution’s and tincture’s injection groups. Lectin injections statistically significantly increased (*p* < 0.001) T lymphocyte count in follicles, compared with physiological solution injections and tincture injections (*p* < 0.05). The T lymphocytes’ quantity was higher in the tincture’s injection group than the physiological solution’s injection group, but there was no statistical significance. There were no statistically significant T lymphocyte quantity changes in the red pulp of the spleen.

The results showed that *Echinacea purpurea* (L.) Moench fresh roots’ tincture suppressed the secondary immune response. According to the literature, purple coneflower fresh roots contain higher quantities of lipophilic alkylamides, which can cause immunosuppression [31]. Opposite to these results, the *Echinacea purpurea* (L.) Moench fresh roots’ lectin injections induced a high secondary immune response, which is typically caused by antigenic proteins. The statistically significant increase in the T lymphocytes’ quantity in PALS and follicles allows us to assume that injections of lectin caused T lymphocytes’ differentiation in the PALS zone of the spleen. Mature T lymphocytes migrate through the follicle zone to neutralize antigens in the peripheral blood circulation. These results of the changes in the spleen’s T lymphocyte confirm blood test results that were previously discussed. Our results do not conflict with other results of lectin immune stimulation in vitro [32,33] and in vivo [34,35].

## 3. Conclusions

To sum up, lectins from purple coneflower fresh roots induced the secondary immune response in the BALB/c mice experimental model in vivo. These results can be supported by a statistically significant neutrophil and monocyte quantity decrease and a statistically significant lymphocyte quantity increase in the blood samples of mice. The vacuolated neutrophils’ quantity could have also been increased because of the lectins’ interaction with the plasmic membranes of the mentioned cells. The statistically significantly higher medium-sized lymphocyte quantity in the blood samples, and the T lymphocyte count in the mice spleens’ PALS zones indicates augmentation of the specific immune response.

Purple coneflower fresh roots’ ethanolic tincture (1:5) causes immune suppression of the specific immune response but has a tendency to induce a primary immune response.

## 4. Materials and Methods

### 4.1. Herbal Material Preparation

Fresh roots (100 g) of the 3-year-old *Echinacea purpurea* L. (Moench) were purchased from the Botanical Garden of Vytautas Magnus University, Kaunas, Lithuania, in November 2015. The specimens were taxonomically identified at the Department of Pharmacognosy of the Lithuanian University of Health Sciences, Kaunas, Lithuania. Fresh roots were frozen at −30 ± 1 °C until extraction of the protein.

### 4.2. Lectin Purification and Identification

LysM lectins were purified and identified in accordance with previous publications of authors [14]. Briefly, fresh EP roots were homogenized with liquid nitrogen in laboratory’s mill. Fine root powder was suspended in phosphate buffer saline (PBS), pH 7.2 (Sigma-Aldrich Chemie GmBh, Steinheim, Germany), supplemented with 2% PVPP in a ratio 1:5 (fine root powder:extraction buffer) and extracted at 4 ºC for 2 h. Extraction was followed by centrifugation at 8500 rpm for 20 min. Obtained supernatant was used for protein precipitation by ammonium sulphate saturation from 60 to 80%. Protein fraction, precipitated by saturating crude’s EP extract with ammonium sulphate (Sigma-Aldrich Chemie GmBh, Steinheim, Germany) from 60 to 80%, was dialyzed against PBS, pH 7.2 buffer, and used for hemagglutinin purification by affinity chromatography method. Mannose-Sepharose affinity column was equipped in purification procedure. Hemagglutinin fraction was eluted with 0.2 M lactose (Sigma-Aldrich Chemie GmBh, Steinheim, Germany) solution in PBS buffer. LysM lectins were identified by liquid chromatography–tandem mass spectrometry. Purified LysM lectins’ fractions were dialyzed against PBS, pH 7.2, filtered through 0.2 μm filter and used in in vivo tests.

### 4.3. Echinacea purpurea (L.) Moench Fresh Roots’ Ttincture Preparation and Analysis

Tincture was made at optimized extraction conditions described in previous publications of authors [36]. *Echinacea purpurea* (L.) Moench fresh roots were homogenized with liquid nitrogen (Achema AB, Jonava, Lithuania) in the cooled IKA M20 universal mill to 0.5–1.5 µm size particles. One part of fine roots’ powder was suspended in five parts of 50% (*v*/*v*) ethanol (Vilniaus degtine AB, Vilnius, Lithuania) and sonicated for 10 min, 30 °C temperature in Elmasonic P ultrasound bath, then centrifuged at 8500 rpm for 10 min in order to separate liquid tincture from residues.

Tincture was concentrated twice with a rotary evaporator and qualitatively–quantitatively analyzed using a high-performance liquid chromatography (HPLC) method, described in European Pharmacopoeia monography 01/2008:1824 [15]. “Waters 2695 Alliance“ chromatography system with “Waters 2998 PDA“ detector was used. Separation was performed using an ACE C18 column (250 mm × 4.6 mm, particle size 5 μm).

Chromatographic peak’s identification was carried out according to the analyte and reference compound retention time, and by comparing UV absorption spectra of reference compounds and analytes, that were obtained by a diode array detector. The purity of the peaks was assessed on the basis of their UV absorption spectra at 200–400 nm. Quantitative assessment of the analytes was performed based on the analytes’ peak area, depending on analytes’ concentration in the test solution. The content of caffeic acid (Sigma-Aldrich Chemie GmBh, Steinheim, Germany) derivates was identified at a wavelength of 320 nm, and quantified by caffeic acid’s equivalents. Tincture’s dry residue was determined by the method described in European Pharmacopoeia [37].

### 4.4. Animals

Male BALB/c mice (8 weeks old, 18–20 g, n = 45) were acquired from Lithuanian University of Health Sciences, Academy of Veterinary, Vivarium. Animals were kept under standard conditions in the Animal Research Center, under optimized hygienic conditions with 12:12 h light–dark cycle. They were fed with a standard pellet diet and water ad libitum. Experiments were approved by State Food and Veterinary Service and Bioethics committee, permission no. G. 2-56.

### 4.5. In Vivo Experiment Model

Three different test animal groups were selected, as follows: Animals (n = 15) in negative control group got 50 μL peritoneal injections of physiological NaCl solution (Sanitas JSC, Kaunas, Lithuania); animals in tincture group (n = 15) got 50 μL peritoneal injection of *Echinacea purpurea* L. (Moench) roots’ tincture (1 μL of tincture contains 200 μg of dry roots’ material). Tincture was made at optimized extraction conditions, described in text previously. Animals in hemagglutinin group (n = 15) received 5 μg (50 μL) of purified hemagglutinin (purification procedure was described previously) via peritoneal injection. Injections were repeated 4 times every 7 days. On 5th week animals were sedated with carbon dioxide and euthanized by cervical dislocation. Spleen samples from each animal were aseptically removed and fixed for 48 h in 10% neutral buffered formalin. Blood samples were collected by cardiac puncture into Microvette® (Sarstedt AG & Co. KG, Nümbrecht, Germany) 200 tubes with EDTA anticoagulant.

### 4.6. Blood Sample Analysis

The peripheral blood samples of each animal were used to measure the total leukocyte count by Neubauer chamber after 1:20 dilution in Turkey’s solution. Diluted blood samples were transferred to the hemocytometer chamber. Calculations were made manually in 25 hemocytometer squares, using a 40× objective lens. Thereafter, blood samples were smeared on a glass slide and stained with May-Grünwald-Giemsa (Sigma-Aldrich Chemie GmBh, Steinheim, Germany) in order to perform differential leucocyte count. Neutrophils, lymphocytes, and monocytes were counted using a light microscope (Nicon, Tokyo, Japan) under a 100× objective lens. Then 300 cells were counted and used for percentage calculations.

### 4.7. Spleen

Formalin-fixed spleen samples were dehydrated and paraffin-embedded. Spleen samples were sectioned into 3-μm-thick pieces and placed on a salinized surface. Sections were deparaffinized with xylene and rehydrated with ethanol, activity of endogenous peroxidases was blocked for immunohistochemical staining, which was previously described by Smalinskiene and others [38]. Briefly, slides were incubated with primary anti-CD3 rabbit/mouse antibodies (DakoCytomation, Glostrup, Denmark), dilution 1:300 for 1 h. Unbound antibodies were washed after finishing incubation with primary antibody, the samples underwent sequential incubations with Advance HRP Link and Advantage HRP Enzyme reagents (Dako, Glostrup, Denmark) for 30 min. Staining was developed with liquid 3,3-diaminobenzidine tetrahydrochloride (DAB) (Sigma-Aldrich Chemie GmBh, Steinheim, Germany) and Substrate Chromogen system (Dako, Glostrup, Denmark).

### 4.8. Evaluation of Spleen’s Sections

Stained spleen samples were scanned with Pannoramic Midi II (3DHistech Kft, Budapest, Hungary) scanner at 0.2325 μm/pxl resolution. For the cell counts, 8 non-overlapping fields (27,392.0 μm^2^ (214.0 μm × 128.0 μm)) in periarterial lymphoid sheaths (PALS), follicles and red pulp were randomly selected. To minimize errors for the measurements of the cell density per μm, the following criteria were used: 1.

Positively stained cells were counted manually by two independent specialists.

### 4.9. Statistical Analysis

The data was expressed as mean ± SD. We performed a one-way analysis of variance (ANOVA) and Wilkinson–Mann–Whitney test, Chi-square test of independence, and Pearson correlation coefficient using SPSS ver. 22.0 (SPSS Inc., an IBM Company, Chicago, IL, USA). A probability *p* < 0.05 was considered to be significant.

## Figures and Tables

**Table 1:** Results of *Echinacea purpurea* (L.) Moench fresh roots’ tincture (1: 5) phenolic acid’s quantity (as equivalent to caffeic acid) determination.

Nr.	RT	Compound	Linear Range, μg/mL	Regression Equation	R^2^	LOD, μg/mL	LOQ, μg/mL	Quantity, μg/mL	Quantity, μg/g, in Fresh Root
1.	4.69	Caftaric acid	1.56–50.00	y = 357,000x – 574,000	0.997	0.158	0.527	1.95 ± 0.10	0.18 ± 0.01
2.	6.83	Chlorogenic acid				0.646	2.154	1.75 ± 0.09	3.16 ± 0.13
3.	7.712	Caffeic acid				0.025	0.085	3.78 ± 0.23	6.80 ± 0.34
4.	9.719	Cichoric acid				0.031	0.104	3.47 ± 0.14	6.24 ± 0.25

RT—retention time, R2—correlation coefficient, LOD—limit of detection, LOQ–limit of quantification.

## Data Availability

The data presented in this study are available in this article.
